# Supplementary material for: Collagen IV and basement membrane at the evolutionary dawn of metazoan tissues
Source: eLife. 2017 Apr 18;6:e24176. doi: 10.7554/eLife.24176 (PMC5395295; doi:10.7554/eLife.24176)
Supplement: Supplementary file 1. — (A) The human collagen IV one gene consists of 52 exons. The number of exons in the Mnemiopsis collagen IV genes ranges from 6 to33. A characteristic feature of collagen IV is the presence of split glycine codons. Eleven of the collagen IV genes possess split glycine codons. (B) Intronic regions are in lowercase and exon regions are in bolded UPPERCASE, underlined nucleotides represent partial codons, Green ‘G’ in the residue column denote glycines encoded by a split codon. DOI: http://dx.doi.org/10.7554/eLife.24176.027 [file elife-24176-supp1.docx]

**Table supplement 1**

**A**

**B**

| **Gene ID** | **Exon Size (bp)** | **Exon Boundaries** |  |
| --- | --- | --- | --- |
| **ML18175a** |  |  |  |
|  | Exon 1 (57bp) | **ATGAAGATC…GGAACTAAG**-gtaaa | MKI…GTK |
|  | Exon 2 (89 bp) | tgcag-**GTCCCTCTC…CACCTAAAG**-tatgt | VPL…HLK |
|  | Exon 3(245 bp) | tccag-**ATCAGTATCC…GCTCGACTAA**-gtagg | SVS…ARL |
|  | Exon 4 (4762 bp) | gcgag-**GAACAGCCG…TGGGTTGGG**-taagt | RTA…WVG |
|  | Exon 5 (111 bp) | cacag-GTACAGCTTC…GCGACAAGT-attac | YSF…CDK |
|  | Exon 6 (63 bp) | tttag-**GACCGGGGAC…GAAGTGAG**-gtaca | TGD…EVS |
|  | Exon 7 (156 bp) | gccag-**TGAGGAGCC…TGGACCGGG**-taagt | EEP…WT**G** |
|  | Exon 8 (143 bp) | ttcag-**GTACAGTTAC…GAGTGTCTTA**-gtaag | YSY…ECL |
|  | Exon 9 (179 bp) | ttcag-**GTCGGGGTT…CGAAAATAG** | **SRG…RK*** |
| **ML16441A** |  |  |  |
|  | Exon 1 (127 bp) | **ATGCGCTG..GGCCCTCAAG-**gtag | MRC..GPQ |
|  | Exon 2 (153 bp) | ccag-**GAGAGGCTG**…**GGTCCAAAAG-**gtaa | **G**EA…GPK |
|  | Exon 3 (108 bp) | acag-**GAGTTCTCG**…**GCGACCTCG-**gtaa | **G**VL..GDL |
|  | Exon 4 (75 bp) | acag-**GTAGTTCTG**…**GGCGTGAAAG-**gtca | **G**SS..GVK |
|  | Exon 5 (126 bp) | ttag-**GAGAGACTG**..**GGACCTCAAG-**tttg | **G**ET..GPQ |
|  | Exon 6 (176 bp) | ccag-**GAGACGCCG**..**GGAGCGAAG-**gtta | **G**DA..GAK |
|  | Exon 7 (124 bp) | tcag-**GGACCAAAA**…**GGAGCTCCAG-**gtac | GPK..GAP |
|  | Exon 8 (165 bp) | aaag-**TACACGTTA**..**GGAAAAGAC-**gtga | VHV..GKD |
|  | Exon 9 (244 bp) | ttag-**ATAATATTC**…**GGTACAGAAG-**gtag | IIF..GEE |
|  | Exon 10 (216 bp) | tcag-**TGCCGCAA**…**GGAGAGAAGG-**gtag | VPQ…GEK |
|  | Exon 11 (126 bp) | ctag-**GAGCTCAA..GGACCTAAAG-**gttc | **G**AQ...GPK |
|  | Exon 12 (189 bp) | tcag-**GTGAACCC..GGACTTGATG-**gtag | **G**EP…GLD |
|  | Exon 13 (184 bp) | tcag-**GACTCAAC…GATGGACT-**gtaa | GLN…DG**L** |
|  | Exon 14 (157 bp) | gcag-**CGACGGACTT..GGAATGGAA-**gtga | DGL..GME |
|  | Exon 15 (198 bp) | ccag-**GGAGAGAAG**..**GGGGCCAAG-**gtga | GEK..GAK |
|  | Exon 16 (208 bp) | tcag-**GGTGAGACA**..**GGAGAACAAG-** gtgac | GET..GEQ |
|  | Exon 17 (176 bp) | acag-**GCCAAAAG**..**GGCGATAAG-**gtgg | **G**QK..GDK |
|  | Exon 18 (144 bp) | caag-**GGAGAGCCT**..**GGAGAACAG-**gtac | GEP..GEQ |
|  | Exon 19 (265 bp) | tcag-**GGTGACCAA**..**ATTGCCAAAG-**gtaca | GDQ..IAK |
|  | Exon 20 (176 bp) | tcag-**GAGCGAAC**..**GGACAGCCG-**gtaa | **G**AN..GQP |
|  | Exon 21 (85 bp) | tcag-**GGAATGGAA.**.**GATGCCCAAG-**gttt | GME..DAQ |
|  | Exon 22 (170 bp) | tcag-**GAGAAAAA**..**GGATCAGTG-**gtaa | **G**EK..GSV |
|  | Exon 23 (171 bp) | acag-**CCAGGAGAA**..**GAGCCTGAG-**gtaa | PGE..EPE |
|  | Exon 24 (225 bp) | ttag-**GTCTCCGAC**..**GGAGCCAAG-**gtga | VSD..GAK |
|  | Exon 25 (135 bp) | tcag-**GGCGAGGAAG**..**GGACCAAAG-**gtta | GEE..GPK |
|  | Exon 26 (129 bp) | tcag-**GGCGTTGCC**..**AGTGATGAG-**gttg | GVA..SDE |
|  | Exon 27 (151 bp) | gcag-**ATCCCCGA**…**CTTGTCAAGG-**tgag | IPE…ACQ |
|  | Exon 28 (131 bp) | ccag-**GTAACGAT**..**CGATGTGCA-**gtaa | **G**TA..RCA |
|  | Exon 29 (108 bp) | ccag-**GCGTGTATG**..**TACCTCATG-**gtca | ACM..YLM |
|  | Exon 30 (153 bp) | acag-**GGTAAGTCT**..**AGAAATCCA-**gtaa | GKS..RNP |
|  | Exon 31 (70 bp) | tcag-**GACCTTACTCA**..**TTGACAGAG-**gtaa | DLT..VDR |
|  | Exon 32 (90 bp) | gcag-**CTGTGGCAC**..**GCAGAGTAA** | AVA..AE* |
| **ML034334a** |  |  |  |
|  | Exon 1 (151 bp) | **ATGGCATCT… TTCATCAAAG**-gtaac | MAS…FIK |
|  | Exon 2 (156 bp) | tccag-**GTAGACAAG … ATCCGAGTAA**- gttaa | GRQ…IRV |
|  | Exon 3 (149 bp) | gggag-**AACCCGGT… GGTCTGAAG**-gtaac | KPG…GLK |
|  | Exon 4 (778 bp) | tttag-**GGAGAGCCC… GTGACCAAGG**-taagt | GEP…GDQ |
|  | Exon 5 (207 bp) | tttag-**GTGAGCCA… GGAGCACAGG**- gtata | **G**EP…GAQ |
|  | Exon 6 (255 bp) | tttag-**GTGAACCT… GGTCCTCAAG**-gtgat | **G**EP…GPQ |
|  | Exon 7 (234 bp) | tgcag-**GAGAACCTG… GGAGTAGAAG**-gtgag | **G**EP…GVE |
|  | Exon 8 (837 bp) | tacag-**GTCTTCCT… GGAGAGAAAG**-gtaat | **G**LP…GEK |
|  | Exon 9 (176 bp) | tccag-**GCGCTCAG… GGAGAACAA**-gtaag | **G**AQ…GEQ |
|  | Exon 10 (253 bp) | ttcag-**GGACAGAAT… GGAATTCCAG**-gtttg | GQN…GIP |
|  | Exon 11 (162 bp) | tctag-**GAGAAGACG… GGACAGAAAG**-gtaag | **G**ED…GQK |
|  | Exon 12 (194 bp) | cccag-**GCGAACAG… GGTGAACAG**-gtaga | **G**EQ…GEQ |
|  | Exon 13 (298 bp) | aacag-**GGAGAAACT… GGACCCCAAG**-gtacg | GET…GPQ |
|  | Exon 14 (101 bp) | ctcag-**GAGATCAG… GGAGAGCCC**-gtacg | **G**DQ…GEP |
|  | Exon 15 (250 bp) | tttag-**GGTGAAAAG… GCAACAAAAG**-gtggg | GEK…ATK |
|  | Exon 16 (242 bp) | ctcag-**GTGTGGCT… GGAGATGCT**-gttag | **G**VA…GDA |
|  | Exon 17 (183 bp) | tgcag-**GGTAACAAC… GGGCCTGAG**-gtatt | GNN…GPE |
|  | Exon 18 (199 bp) | tttag-**GGACCTGCT… GGACCTGCTG**-gtacg | GPA..GPA |
|  | Exon 19 (176 bp) | tacag-**GCGAGAGAG… ACTACTGAA**-gtaag | **G**ER…TTE |
|  | Exon 20 (103 bp) | atcag-**GAACCTGAT… GGAAGCGCT**-ggtaa | EPD…GSA |
|  | Exon 21 (287 bp) | tacag-**GTTCCTGTC… TATCTCATG**-gtaag | GSC…YLM |
|  | Exon 22 (267 bp) | cgtag-**GTTACTTCA… ACCATCTAA** | VTS…TI* |
| **ML18198a** |  |  |  |
|  | Exon 1 (145 bp) | **ATGGCATCT… TTTGTAAAAG**-gtaaa | MAS…FVK |
|  | Exon 2 (152 bp) | tcagg-**TAGACAAG… GGAGCCCGG**-gtaaa | **G**RQ…GAR |
|  | Exon 3 (83 bp) | ctcag-**ACGGACTTC… GTGGCCAG**-tattt | TDF…VA**S** |
|  | Exon 4 (650 bp) | atcag-**CAACGAT… GGTGATAACG**-gtgag | NDC…GDN |
|  | Exon 5 (195 bp) | ttcag-**GAGAGCCA… GGAGAACCAG**-gtaac | **G**EP…GEP |
|  | Exon 6 (255 bp) | ttcag-**GTGAACCC… GGAGAAAAAG**-gttag | **G**EP…GEK |
|  | Exon 7 (234 bp) | tccag-**GTAATCCT… GGCCCACCAG**-gtaag | **G**NP…GPP |
|  | Exon 8 (849 bp) | tctag-**GCAAACGT… GGAGAAAACG**-gtaag | **G**KR…GEN |
|  | Exon 9 (176 bp) | ctcag-**GTGCCCCC… GGAGATAAG**-gtaaa | **G**AP…GDK |
|  | Exon 10 (256 bp) | tcaag-**GGAGCACCT… GGTCAGCCTG**-gttag | GAP…GQP |
|  | Exon 11 (165 bp) | atcag-**GACCAAACG… GGCTCCAAAG**-gtatc | **G**PN…GSK |
|  | Exon 12 (197 bp) | cccag-**GTGAGCCT… GCACCTCAG**-gttag | **G**EP…APQ |
|  | Exon 13(301 bp) | atcag-**GGGCCAAC… GGTCTTCCAG**-gtaca | **G**TP…GLP |
|  | Exon 14 (101 bp) | ttcag-**GAGATGAA… GGAGAACAG**-gtcag | **G**DE…GEQ |
|  | Exon 15 (265 bp) | ggaga-**ACAGGTCA… GGTACTCCTG**-gtcag | **G**EK…GTP |
|  | Exon 16 (248 bp) | tatag-**GTGAGCCG… GGAGAACCC**-gtacg | **G**EP…GEP |
|  | Exon 17 (183 bp) | ctcag-**GGCGAACCT… GGACCGCCA**-gtatg | GEP…GPP |
|  | Exon 18 (205 bp) | cccag-**GGAGCAGTA… CAGCCTAATG**-gtggg | GAV…QPN |
|  | Exon 19 (179 bp) | cccag-**ATGAAAAG… ACTTCTGAT**-gtaag | DEK…TSD |
|  | Exon 20 (103 bp) | ttcag-**ATTCCTGAT… GGAACCACAG**-gtaaa | IDP…GTT |
|  | Exon 21 (287 bp) | tccag-**GTTCCTGTA… TTCTTACAG**-gtatt | **G**SC…FLQ |
|  | Exon 22 (267 bp) | aacag-**GCTACCTCA… TCTGCGTAA** | ATS…SA* |
|  |  |  |  |
| **ML18197a** |  |  |  |
|  | Exon 1 (142 bp ) | **ATGGCATCT…TTCGTGAAAG**-gtggg | MAS…FVK |
|  | Exon 2 (152 BP) | tttag-**GCAGACCG…GGTTTCCGC**-gtaag | **G**RP…GFR |
|  | Exon 3 (141 BP) | tacag-**GGTGAACCT…GGGGACAGG**-gtaag | GEP…GDR |
|  | Exon 4 (640 bp) | cttag-**GGAGCACGA… GGCGCTCCAG**-gtaaa | GAR…GAP |
|  | Exon 5 (210 bp) | ttcag-**GCGAAGAC… GGAGCTCAGG-**gtttg | **G**ED…GAQ |
|  | Exon 6 (255 bp) | ttcag-**GTCCCAGG… GGTTCTCAAG**-gtatg | **G**PR…GSQ |
|  | Exon 7 (234 bp) | ccaag-**GTGAAAGTG… GGTCCCGAAG**-gtaaa | **G**ES…GPE |
|  | Exon 8 (840 bp) | aacag-**GACCGAAA… GGACTTAAAG**-gtgag | **G**PK…GLK |
|  | Exon 9 (176 bp) | accag-**GTAGCATA… GGTCCCAAG**-gtttg | **G**SI…GPK |
|  | Exon 10 (259 bp) | tgcag-**GGAGAGCCT… GGTCCTAGAG**-gtgag | GEP…GPR |
|  | Exon 11 (165 bp) | cccag-**GAGAGCCA… GGGGAGCCAG**-gtttg | **G**EP…GEP |
|  | Exon 12 (194 bp) | tgcag-**GTGAGAAA… GGAATAAAG**-gttgg | **G**EK…GIK |
|  | Exon 13 (258 bp) | ctcag-**GGTGAGGTT… CAGGGTGTG**-gtagg | GEV…QGV |
|  | Exon 14 (262 bp) | tttag-**GGTGAAACT… GGCCAACCAG**-gttag | GET..GQP |
|  | Exon 15 (245 bp) | cccag-**GTGAACCT… GGAGAACCT**-gtaag | **G**EP…GEP |
|  | Exon 16 (183 bp) | ctcag-**GGATCACCA… GGACCCGCG**-gtaag | GSP…GPA |
|  | Exon 17 (199 bp) | cccag-**GGACCTGCG… GGAGAAAACG**-gtgcg | GPA…GEN |
|  | Exon 18 (287 bp) | tccag-**GTTCCTGC…TATGCCATG**-gtaag | **G**SC…YAM |
|  | Exon 19 (273 bp) | ctcag-**GTGTCATCA… GAAGAGTGA** | VSS…EE* |
| **ML034337a** |  |  |  |
|  | Exon 1 (132 bp) | **ATGGCAAGC…TGCCGTAGG**-gtgag | MAS…CRR |
|  | Exon 2 (73 bp) | tacag-**AGATCACGA…GGTCACGCAG**-gtatg | RSR…GHA |
|  | Exon 3 (251 bp) | ccgca-**GGACCGATG…GGTCCTACT**-gtaag | **G**PM…GPT |
|  | Exon 4 (142 bp) | tctag-**GGACTTACC…GGAGAAACAG**-gttaa | GLT…GET |
|  | Exon 5 (81 bp) | aacag-**GAGCCACC…GGAGAAAAAG**-gtgaa | **G**AT…GEK |
|  | Exon 6 (135 bp) | cttca-**GGAATCCAA…GGACCGAAGG**-gtaag | **G**IQ…GPK |
|  | Exon 7 (248 bp) | cttag-**GTGAGGCT…GGAGAAACT**-gtgag | **G**EA…GET |
|  | Exon 8 279 bp) | ttcag-**GGAGACCAA…GGAGACCAG**-gtata | GDQ…GDQ |
|  | Exon 9 (100 bp) | ttcag-**GGAGAGACT…GGACCCCCAG**-gtatt | GET…GPP |
|  | Exon 10 (255 bp) | tttag-**GAGAAAAC…GGAGAAACAG**-gtcag | **G**EN..GET |
|  | Exon 11 (71 bp) | tgcag-**GCGAGAAG…GGTGTGAAT**-gtaag | **G**EK…GVN |
|  | Exon 12 (163 bp) | gtagg-**GAGAGGTC…GGGATTCAAG**-gtgag | GEV…GIQ |
|  | Exon 13 (1122 bp) | tgcag-**GAGACGAA…GGTGAAACAG**-gtaaa | **G**DE…GET |
|  | Exon 14 (89 bp) | tttta-**GGTGCAAAA…GGAGCGGAG**-gttag | GAK…GAE |
|  | Exon 15 (208 bp) | cttag-**GGAGAAGTT…GGAGAGAAAG**-gtaat | GEV…GEK |
|  | Exon 16 (176 bp) | attag-**GACAGCCC…GGAGATCAG**-gttag | **G**QP…GDP |
|  | Exon 17 (144 bp) | cccag-**GGAGAAACC…GGAGATCAG**-gtaca | GET…GDQ |
|  | Exon 18 (265 bp) | ttcag-**GGTGAAAAA…ATCGCCAAAG**-gtaag | GEK…IAK |
|  | Exon 19 (167 bp) | tacag-**GAGCCAAC… GGAGATAAG**-gtttg | **G**AN…GDK |
|  | Exon 20 (97 bp) | cgcag-**GGTCCACAA…GATGCTAAAG**-gtaaa | GPQ…DAK |
|  | Exon 21 (170 bp) | ataca-**GGAGAAGCC… GGAGCGACG**-gtgag | **G**EA…GAT |
|  | Exon 22 (165 bp | atcag-**GGTGAAACT… CAATTGCCT**-gtaag | GET…QLP |
|  | Exon 23 (100 bp) | ttcag-**CCTGATGAT… GGAGAACAAG**-gtcgg | PDD…GEQ |
|  | Exon 24 (144 bp) | aacag-**GTATTGAA… GGAGAAACTG**-gtgcg | **G**IE…GET |
|  | Exon 25 (380 bp) | ttata-**GGAGATACT… ATGATGCAG**-gtatg | **G**DT…MMQ |
|  | Exon 26 (119 bp) | cccag-**TGTTCCGGT… CTTATCGGG**-tatgt | CSS…LI**G** |
|  | Exon 27 (127 bp) | ctcag-**GTACGTCTCA… TATCTTATG**-gtgag | YVS…YLM |
|  | Exon 28 (294 bp) | tccag-**GTAACGTCC… CAAGATTAA** | VTS…QD* |
| **ML034336a** |  |  |  |
|  | Exon 1   (154 bp) | **ATGGCATCT… TTCGTGAAAG**-gttcg | MAS…FVK |
|  | Exon 2   (152 bp) | tacag-**GTAGACCC… GGTTTCCGT**-gtaag | **G**RP…GFR |
|  | Exon 3   (153 bp) | cacag-**GGAGAGAAC… GGGGCACC**-gtaag | GEN…GAP |
|  | Exon 4   (141 bp) | gttag-**GGCGAGCCT… GGTGACAAG**-gtcag | GEP…GDK |
|  | Exon 5   (637 bp) | aacag-**GGAGCAACT… GGAGCTCAAG**-gtaac | GAT…GAQ |
|  | Exon 6   (207 bp) | aacag-**GTGACATT… GGCGAGCCAG**-gttcg | **G**DI…GEP |
|  | Exon 7   (255 bp) | gtcag-**GTGACAGG… GGACCCACCG**-gtaac | **G**DR…GPT |
|  | Exon 8   (198 bp) | tccag-**GGGAAACT… GGAGTTCAGG**-gtgaa | **G**ET…GVQ |
|  | Exon 9   (837 bp) | ttcag-**GGCCCAAA… GGAGTCAAAG**-gtaat | **G**PK…GVK |
|  | Exon 10 (176 bp) | tctag-**GAGTGAAA… GGAGTCACT**-gtgag | **G**VK…GVT |
|  | Exon 11 (253 bp) | catag-**GGGGAGGCT… GGTGAAAGAG**-gtaaa | GEA…GER |
|  | Exon 12 (162 bp) | tcaag-**GTATTGAT… GGAGAAAAGG**-gtttg | **G**ID…GEK |
|  | Exon 13 (194 bp) | tgtag-**GAGACAAG… GGAATCAAA**-gtaag | **G**DK…GIK |
|  | Exon 14 (298 bp) | tttag-**GGCGAAGCT… GGACCCAAGG**-gtatg | GEA…GPK |
|  | Exon 15 (101 bp) | cacag-**GAATTCAG… GGAGAAAAG**-gtggg | **G**IQ…GEK |
|  | Exon 16 (250 bp) | tcaag-**GGTGAGCTT… GCTGAACCAG**-gtata | GEL…AEP |
|  | Exon 17 (242 bp) | ccaag-**GTGATAAC… GGAGAAGCT**-gtgag | **G**DN…GEA |
|  | Exon 18 (183 bp) | atcag-**GGAATGCCA… GGCCCACAG**-gttta | GMP…GPQ |
|  | Exon 19 (199 bp) | cctag-**GGACCCGAA… GGAGACAAAG**-gtatt | GPE…GDK |
|  | Exon20 (287 bp) | ctgca-**GGTTCCTG… TACGTTATG**-gtggg | **G**SC…YVM |
|  | Exon 21 (273 bp) | cgcag-**GTCACTTCA… GACTTGTAA** | VTS…DL* |
| **ML17503a** |  |  |  |
|  | Exon1(140bp) | **ATGTTGCAT..GGAAGAGAT**-gtga | MLH…YYG |
|  | Exon2(116bp) | tcag-**GGCACTGAC..GGAGAAAAGG**-gtta | GNT…GTR |
|  | Exon3(129bp) | gccag-**ACTTGACAG..GGACGTCCAGG**-tagt | DLT…GRP |
|  | Exon4(155bp) | tatag-**CTATGACA..GGTCGAGAC**-gtgag | AMT…GRD |
|  | Exon5(153bp) | tccag-**GGGAGGCCA..GGCTTCAGG**-gtgag | GRP…GFR |
|  | Exon6(180bp) | accag-**GGAGAAAAC..GGAGAACCG**-gtaag | GEN…GEP |
|  | Exon7(144bp) | gtcag-**AAAGGATCT..GGAAACAAT**-gtgag | KGS…GNN |
|  | Exon8(127bp) | cccag-**GGTATGCCA..GGACGACCAG**-gtaaa | GMP…GRP |
|  | Exon9(123bp) | catca-**GGAAATGC..GGTAACAGAG**-gtgag | GNA…GNR |
|  | Exon10(65bp) | ccaag-**ATCCTTTT..CAACAAAAG**-gtgag | DPF…QQK |
|  | Exon11(195bp) | tgcag-**GGATTGCCA..GATGGCATG**-gtaag | GLP…DGM |
|  | Exon12(171bp) | tttag-**AGAGCAAGA..GGACCGAGG**-gtatg | RAR…GPR |
|  | Exon13(234bp) | tccag-**GGAACAGAT…GGAGAACGA**-gtgag | GTD…GER |
|  | Exon14(282bp) | tgcag-**GGAGTACCA…GGTCTACCA**-gtaag | GVP…GLP |
|  | Exon15(187bp) | cccag-**GGTCGACCC..GGCTATCCAG**-gtaag | GRP…GYP |
|  | Exon16(113bp) | tctca-**GGAAGAGAT..GGGAAAAAT**-gttag | GRD…GKN |
|  | Exon17(88bp) | aggga-**ATTCCGGGA..GGAAGTCCAG**-gtgga | GIP…GSP |
|  | Exon18(153bp) | ttcca-**GGCGAAGTA..TGTCCGAGTG**-gtaag | GEV…CPS |
|  | Exon19(113bp) | tttta-**GGTTCGGTT..TTGATGATG**-gtaag | GSV…LMM |
|  | Exon20(100bp) | cacag-**GGGCGAGAC..GTCATGTCCG**-gtatg | GRD…VMS |
|  | Exon21(93bp) | cttta-**GGCTCTAGT..GGTGCCATAG**-gtgag | GSS…GAI |
|  | Exon22(138bp) | ccgca-**GGAATTTTC..CAACTTTAA** | GIF…QL* |
| **ML17504a** |  |  |  |
|  | Exon 1 (40 bp) | **ATGGTGTCG…TTGCAACTAT**-gtaag | MVS…LQL |
|  | Exon 2 (143 bp) | tatag-**ACCAAGAA…GGCGAAGCT**-gtaag | **Y**QE..GEA |
|  | Exon 3 (145 bp) | tacag-**GGAGCTACA…GGCCGACCAG**-gtaat | GAT…GRP |
|  | Exon 4 (125 bp) | atcag-**GAAGACCA…GGAGAACCG**-gtgag | **G**RP…GEP |
|  | Exon 5 (181 bp) | atcag-**GGAGACCCC…GGACGTCCGG**-gttag | GDP…GRP |
|  | Exon 6 (125 bp) | tccag-**GAAGTATT…GGAGAAATG**-gtaag | **G**SI…GEM |
|  | Exon 7 (117 bp) | ttcag-**GGGAGACCA…GGAAAAAAT**-gtgag | GRP…GKN |
|  | Exon 8 (153 bp) | cccag-**GGATTACCT…GGTCTTCCT**-gtggg | GLP…GLP |
|  | Exon 9 (90 bp) | cgcag-**GGAGAAGAT…GGATTCCCG**-gtatg | GED…GFP |
|  | Exon 10 (64 bp) | tccag-**GGCACACCG…GGTATCCCAG**-gtaat | GTP…GIP |
|  | Exon 11 (116 bp) | tgcag-**GAAACAGG…GGAACAACT**-gtaag | **G**NR…GTT |
|  | Exon 12 (106 bp) | ggcag-**AATTTACCA…GGAATGAGAG**-gtatt | NLP…GMR |
|  | Exon 13 (78 bp) | ctcag-**GTACTCCTG…GGACTTCCAG**-gtaat | **G**TP…GLP |
|  | Exon 14 (80 bp) | ctcag-**GTATGAGAG…GGAATAAAT**-gtaag | **G**MR…GIN |
|  | Exon 15 (163 bp) | tgcag-**GGTATAGAC…GGCACTTCAG-**gtatt | GID…GTS |
|  | Exon 16 (153 bp) | gccag-**GTATCGAT…GGCCGAAAAG**-gtacg | **G**ID…GRK |
|  | Exon 17 (132 bp) | gccag-**GTGAGACGG…ACCTTACTTA**-gttag | **G**ET…TLL |
|  | Exon 18 (140 bp) | tccag-**GTACGGAC…TTTACTTAC**-gtgag | STD…FTY |
|  | Exon 19 (127 bp) | tcagg-**GGCCTCGTG…GGAAATCCAG**-gtaaa | GPR…GNP |
|  | Exon 20 (108 bp) | cccag-**GGCTACCC…GGTTTCAGAG**-gttag | **G**LP…GFR |
|  | Exon 21 (131 bp) | catag-**GAGATCCA…GGGCAACCG**-gtaag | **G**DP…GQP |
|  | Exon 22 (90 bp) | tgcag-**GGAACTCCA…GGTTATCAG**-gtaca | GTP…GYQ |
|  | Exon 23 (144 bp) | ttcag-**GGAAACAGA…GGGAAGAGG**-gtttg | GNR…GKR |
|  | Exon 24(141 bp) | cccag-**GGTGAACCG…GGACCTCCT**-gtacg | GEP…GPP |
|  | Exon 25 (141 bp) | ctcag-**GGGCCCCCT…CTCAGGGGC**-cccct | GPP…GLP |
|  | Exon 26 (300 bp) | ctcag-**GGAAGGCCG…GGCACCCCG**-gtaat | GRP…GTP |
|  | Exon 27 (114 bp) | ttcag-**GGTCTGCCA…GTTATGACG**-gtaat | GLP…VMT |
|  | Exon 28 (166 bp) | ctcag-**GGCACTGGA…AGAATAAACA**-gtata | GTG…RIN |
|  | Exon 29 (188 bp) | tacag-**GGATGTCTA…GGCTGCGAG**-gtaag | RMS…GCE |
|  | Exon 30 (117 bp) | tgcag-**GTCAAGTTT…CCGACTGCG**-gtaat | VKF…PTA |
|  | Exon 31 (165 bp) | ttcag-**GTAACAAAC…CTTATCATG**-gtaaa | VTN…LIM |
|  | Exon 32 (59 bp) | tacag-**GCTAAAGAT…TGCATGAG**-gtaaa | AKD…CMR |
|  | Exon 33(199 bp) | **TTCAGGAGGTTCTC…ATTTTATAA** | RFS…IL* |
| **ML17502a** |  |  |  |
|  | Exon 1 (438 bp) | **ATGCCTGGT..GGAAGAGAT-**gtga | MPG…GRD |
|  | Exon 2 (97 bp) | tcag-**GGCACTGAC**..**GGAGAAAAGG**-gtta | GTD…GEK |
|  | Exon 3 (146 bp) | ttca-**GGTGAGCCA**..**GGACAGAAG-**gttt | **G**EP…GQK |
|  | Exon 4 (214 bp) | ttca-**GGAGAAAAT**..**GGAGACAAA-**ggta | GEN…GDK |
|  | Exon 5 (230 bp) | tcag-**GGAAGCAGC**..**GGATCTCCG-**gtaa | GSS…GSP |
|  | Exon 6 (411 bp) | ccag-**GGTTTGAAC**.. **GGTGATAAG-**gttg | GLN…GDK |
|  | Exon 7 (285 bp) | caag-**GGTAACTCT**.. **GGAAACGAT-**gtta | GNS…GND |
|  | Exon 8 (456 bp) | gcag-**GGACAGAAT**.. **GGAACTGAT-**gtta | GQN…GTD |
|  | Exon 9 (288 bp) | ttag-**GGCGAACCT**.. **GGAGCACCT-**gtaa | GEP…GAP |
|  | Exon 10 (291 bp) | tcag-**GGAGTTTC**.. **GGTAACCCG-gtca** | GVS…GNP |
|  | Exon 11 (313 bp) | tcag-**GGAAGTCCA**.. **GGTTCCCCA-**ggta | GSP...GSP |
|  | Exon 12 (203 bp) | gcag-**GCGCTCCGG**.. **GGACCCCCT-**gtga | GAP…GPP |
|  | Exon 13 (133 bp) | ctag-**GGCCCAGCA**.. **GGACCCCCCG**-gtta | GPA…GPP |
|  | Exon 14 (156 bp) | ccag-**GACCACAAG**.. **TTTATGGAC-**ggta | **G**PQ…FMD |
|  | Exon 15 (133 bp) | acag**GCGGTTCCA**.. **CAAACTGCG-**ttag | GGS…QTA |
|  | Exon 16 (193 bp) | acag-G**TGGTTGGCT**.. **CCATACGAG-**gtca | WLA…PYE |
|  | Exon 17 (148 bp) | ccag-**GTTGCATGT**.. **GTAACATCTG**-gttt | VAC…VTS |
|  | Exon 18 (134 bp) | tcca-**GAATCGGCA**.. **TCTGGACAG-**gtaa | ESA…SGQ |
|  | Exon 19 (138 bp) | tcag-GTAGCAGAC.. **AAAATTTAA** | VAD…KI* |
| **ML17501a** |  |  |  |
|  | Exon 1 (120 bp) | **ATGAATTA…GGTGCTCGG-**gtaag | MKL…GAR |
|  | Exon 2 (108 bp) | actag-**ACAGCAAGT**…**GGAGATCGG-**gtaag | TAS…GDR |
|  | Exon 3 (307 bp) | ttcag-**GGTCCCGCA**…**GGAGAAAGTG-**gtagg | GPA…GES |
|  | Exon 4 (278 bp) | cgtag-**GTCGAATA**…**GGAAAAGAT-**gtggg | **G**RI…GKD |
|  | Exon 5 (97 bp) | tgcag-**GGAACTCCC**… **GGTGAAAAAG**-gtgcg | GTP…GEK |
|  | Exon 6 (146 bp) | tgcag-**GTGACCGC**…**GGAGCTCAA-**gtaag | **G**DR…GAQ |
|  | Exon 7 (109 bp) | tttag-**GGAGACAGA**…**GGGACGAAAG**-gttta | GDR…GTK |
|  | Exon 8 (186 bp) | tccag-**GAGAAAGA**… **GGAGCACCAG**-gcaag | **G**ER…GAP |
|  | Exon 9 (149 bp) | ttcag-**GACTGATG**…**GGGACACCC-**gtaag | **G**LM…GTP |
|  | Exon 10 (208 bp) | accag-**GGATTACCC**…**GGAGAGAGAG**-gtcag | GLP…GER |
|  | Exon 11 (203 bp) | ttaag-**GAAGCCAG**…**GGTGAAAAG-**gttcc | **G**NA…GDK |
|  | Exon 12 (127 bp) | accag-**GGAAACGCT**…**GGAGACAAGG**-gtaag | GNA…GDK |
|  | Exon 13 (158 bp) | tacag-**GTGAGGCA**…**GGTAACGAT-**gtgag | **G**EA…GND |
|  | Exon 14 (456 bp) | tacag-**GGAAGGAAC**…**GGCGAAAAG-**gtaag | GRN…GEK |
|  | Exon 15 (288 bp) | ttcag-**GGTGAACCA**…**GGTCGTCCG-**gttag | GEP…GRP |
|  | Exon 16 (291 bp) | tttag-**GGAGATTCC…GGCAGAGAT-**gtcag | GDS…GRD |
|  | Exon 17 (516 bp) | ttcag-**GGAACTCCC…GGACCT ACA-**gtaag | GTP…GPT |
|  | Exon 18 (151 bp) | aatag-**GGGGAAGCA…GGGCGTGCAG**-gtaaa | GEA…GRA |
|  | Exon 19 (138 bp) | tacag-**GCGCGCCA…TTTGTAGATG**-gtaag | **G**AP…FVD |
|  | Exon 20 (130 bp) | acaag-**GAGACTCC…AAGACAAC-**gtgag | **G**DS…KTT |
|  | Exon 21 (59 bp) | ttcag-**TTGGTTGGCC…ATGCCAAGAG**-gtcag | WLA…MPR |
|  | Exon 22 (130 bp) | tttag-**GTAAACTG**…**CCAGAAGg-**gttag | **G**KL…PEG |
|  | Exon 23 (100 bp) | ttcag-**AACCCCAACT**…**CGAAAATAG** | TPT…RK* |
| **ML047918a** |  |  |  |
|  | Exon 1 (133 bp) | **ATGCTCTGC…ACGGCCGGAG**-gtaag | MLC…TAG |
|  | Exon 2 (173 bp) | tccag-**ATTTCCGA…AAAGCTTTC**-gtaag | DFR…KAF |
|  | Exon 3 (137 bp) | tttag-**GACGGCTCC…TGGAACAG**-taaaa | DGS…WNR |
|  | Exon 4 (169 bp) | tgcag-**GAAGTGGAAC…TTCATTTCG**-gtatg | KWN…FIS |
|  | Exon 5 (102 bp) | tgcag-**GCAGGTCAG…CAGTGCAAG**-gtaag | AGQ…QCK |
|  | Exon 6 (192 bp) | ttcag-**AGTCACCAA…TTCAAATAG** | SHQ…FK* |
